# Functionality and Quality of Asthma mHealth Apps and Their Consistency With International Guidelines: Structured Search and Evaluation

**DOI:** 10.2196/47295

**Published:** 2024-01-10

**Authors:** Billy Robinson, Eleni Proimos, Daniel Zou, Enying Gong, Brian Oldenburg, Katharine See

**Affiliations:** 1 Department of Respiratory Medicine Northern Health Epping Australia; 2 Faculty of Medicine, Nursing and Health Sciences Monash University Melbourne Australia; 3 Faculty of Medicine, Dentistry and Health Sciences University of Melbourne Melbourne Australia; 4 School of Population Medicine and Public Health Chinese Academy of Medical Sciences and Peking Union Medical College Beijing China; 5 Baker Heart and Diabetes Institute Melbourne Australia; 6 Academic and Research Collaborative in Health LaTrobe University Melbourne Australia

**Keywords:** asthma, mobile health, mHealth, app, mobile, chronic disease, systematic review, smartphone, review methodology, respiratory, compliance, guideline, guidelines, review of apps, evaluation, quality, best practices, apps, mobile phone

## Abstract

**Background:**

Asthma is a chronic respiratory disorder requiring long-term pharmacotherapy and judicious patient self-management. Few studies have systematically evaluated asthma mobile health (mHealth) apps for quality and functionality; however, none have systematically assessed these apps for their content alignment with international best practice guidelines.

**Objective:**

This review aims to conduct a systematic search and evaluation of current mHealth apps in the Australian marketplace for their functionality, quality, and consistency with best practice guidelines.

**Methods:**

The most recent Global Initiative for Asthma (GINA) guidelines were reviewed to identify key recommendations that could be feasibly incorporated into an mHealth app. We developed a checklist based on these recommendations and a modified version of a previously developed framework. App stores were reviewed to identify potential mHealth apps based on predefined criteria. Evaluation of suitable apps included the assessment of technical information, an app quality assessment using the validated Mobile App Rating Scale (MARS) framework, and an app functionality assessment using the Intercontinental Medical Statistics Institute for Health Informatics (IMS) Functionality Scoring System. Finally, the mHealth apps were assessed for their content alignment with the GINA guidelines using the checklist we developed.

**Results:**

Of the 422 apps initially identified, 53 were suitable for further analysis based on inclusion and exclusion criteria. The mean number of behavioral change techniques for a single app was 3.26 (SD 2.27). The mean MARS score for all the reviewed apps was 3.05 (SD 0.54). Of 53 apps, 27 (51%) achieved a total MARS score of ≥3. On average, the reviewed apps achieved 5.1 (SD 2.79) functionalities on the 11-point IMS functionality scale. The median number of functionalities identified was 5 (IQR 2-7). Overall, 10 (22%) of the 45 apps with reviewer consensus in this domain provided general knowledge regarding asthma. Of 53 apps, skill training in peak flow meters, inhaler devices, recognizing or responding to exacerbations, and nonpharmacological asthma management were identified in 8 (17%), 12 (25%), 11 (28%), and 14 (31%) apps, respectively; 19 (37%) apps could track or record “asthma symptoms,” which was the most commonly recorded metric. The most frequently identified prompt was for taking preventive medications, available in 9 (20%) apps. Five (10%) apps provided an area for patients to store or enter their asthma action plan.

**Conclusions:**

This study used a unique checklist developed based on the GINA guidelines to evaluate the content alignment of asthma apps. Good-quality asthma apps aligned with international best practice asthma guidelines are lacking. Future app development should target the currently lacking key features identified in this study, including the use of asthma action plans and the deployment of behavioral change techniques to engage and re-engage with users. This study has implications for clinicians navigating the ever-expanding mHealth app market for chronic diseases.

**Trial Registration:**

PROSPERO CRD42021269894; https://www.crd.york.ac.uk/prospero/display_record.php?RecordID=269894

**International Registered Report Identifier (IRRID):**

RR2-10.2196/33103

## Introduction

### Background

Asthma is a chronic respiratory disorder that is clinically defined as a combination of typical episodic respiratory symptoms, such as wheezing, shortness of breath, cough, chest tightness, and significant variable reversible airflow limitation [1]. When the frequency or severity of these symptoms increases compared with the baseline respiratory status, it represents an “asthma exacerbation” or “flare-up” [2]. Judicious self-monitoring and management of regular asthma medications, symptoms, and exacerbations are key to allowing patients with asthma to live with a high quality of life and prevent hospitalizations or death [3]. The Global Initiative for Asthma (GINA) regularly releases updated best practice asthma guidelines based on reviews of scientific literature by an international panel of experts [4]. Many local asthma management guidelines have been derived from these international guidelines. In addition to pharmacotherapy, the guidelines advise that patient education on medication adherence, exacerbation recognition, and management is key to self-management [4].

Asthma is a significant chronic health issue worldwide, affecting 1% to 18% of the global population [4]. Australia is no exception, with asthma affecting millions and accounting for 34% of Australia’s respiratory disease burden and 2.5% of the total disease burden [5]. Asthma leads to numerous emergency department visits and urgent health care visits [5]. Furthermore, those living with asthma report a poorer quality of life and are less likely to rate their health status as excellent or very good [5]. When observing the total cost that asthma has on the Australian health system, it is evident that hospital-related costs outweigh non–hospital-related costs (Aus $205 million/year [approximately US $150 million] vs Aus $163 million/year [approximately US $120 million]) [5]. Theoretically, reducing exacerbations would reduce the requirement for hospitalizations; unplanned primary care presentations; and indirect costs, such as work absenteeism, and thus assist in reducing these costs.

With the increasing availability of smartphones, mobile health (mHealth) apps have become accessible to a large percentage of the population and represent a potential medium through which patients can improve their ability to self-manage asthma. Deloitte’s recent review of Australia’s telecommunication status found that 89% of the Australian population uses smartphones [6]. These apps are already available for download and use; however, it is imperative that a review of their quality, functionality, and alignment with evidence-based best practices is conducted to inform both users and health professionals. These apps represent an opportunity for new ways to empower patients to track asthma symptoms, learn about their condition, and undertake practical self-management strategies. The established Mobile App Rating Scale (MARS) is generally used to assess the usability and overall quality of mHealth apps [7,8]. Although systematic evaluations of asthma mobile apps have been conducted in the past, many of these studies did not assess the apps’ functionality or quality using a validated rating scale, such as MARS [9-11]. Furthermore, to our knowledge, none of these prior evaluations assessed all available apps systematically for the presence and quality of information they provide compared with available best practice management guidelines, such as the GINA guidelines [9-11].

This systematic search and evaluation review assessed the functionality and quality of free and paid asthma mHealth apps targeted toward adults with asthma available from the Apple App Store (iOS) and Google Play Store (Android), as well as their consistency with recommendations from the GINA guidelines, making it the first review of its kind.

### Objective

The objective of this review was to conduct a systematic search of available English-language mHealth apps targeted toward adults with asthma in Australia, to evaluate their overall quality and functionality and to assess the consistency and quality of the content and information they provide in alignment with current best practice guidelines for asthma management.

## Methods

### Overview

The GINA guidelines were reviewed by 2 medical professionals to identify and establish a consensus of key recommendations from the guidelines that could feasibly be incorporated into an app for asthma management. The mobile apps in the selected app stores were identified and screened based on the selection criteria. Finally, we assessed the quality, functionality, and alignment of the apps with the guidelines identified in the first step of the screened mHealth apps. An in-depth description of the research protocol was published the previous year [12].

### Study Setting

Given the primary residences of the researchers involved in this review, this study was conducted by medical practitioners, medical students, and digital health researchers using apps available in Australia. The mHealth apps presented in English on Australian mobile app stores were assessed. Some mHealth apps identified in this review may not be available in regions outside Australia. Similarly, apps available in other regions may not be available in Australia. However, given that most of the apps identified in this review are also available in other English-speaking regions such as the United Kingdom and the United States, the results are largely generalizable to these regions. Given that the researchers were adult medical practitioners who did not manage pediatric patients, only those mHealth apps targeted toward adults with asthma were evaluated.

Wherever possible, the PRISMA (Preferred Reporting Items for Systematic Reviews and Meta-Analyses) guidelines for systematic reviews were followed [13]. Given that this was a review of mobile apps instead of journal articles, some items in the PRISMA checklist were not relevant to this review. The checklist is shown in Multimedia Appendix 1.

### Review of the GINA Guidelines and Checklist Creation

To assess the usability and overall quality of the app, we used the established MARS [7,8]. A review of the available literature using the CINAHL, MEDLINE, Embase, and PubMed databases revealed that 1 research group had developed an asthma app assessment framework yet to be derived and validated into an instrument [14]. For the reasons outlined in our published research protocol, we decided to combine aspects of the framework by Guan et al [12] with our own checklist derived from the GINA guidelines. Two reviewers, BR and KS, independently assessed the 2020 GINA guidelines for identifiable recommendations that could be incorporated into an mHealth app. Following this, the reviewers examined each other’s identified recommendations to see whether a consensus could be reached on the recommendations from the GINA guidelines that could be incorporated into an mHealth app. The identified recommendations from each author and those where a consensus was reached, which represent the final identified recommendations, are shown in Table 1.

A final checklist modified from the framework by Guan et al [14] (Table 1) was developed to include recommendations we identified from the above process while excluding the information we gathered through the MARS framework. To determine app consistency with the GINA guidelines, participants were assessed for the presence or absence of features identified through this process. This is further discussed in subsequent sections.

**Table 1:** Recommendations identified from the Global Initiative for Asthma guidelines that could be incorporated into a mobile health app.

Reviewer 1	Reviewer 2	Consensus reached
Assess symptom control (eg, ACQ^a^)	Support for assessing symptom control for a 4-week period	Support for assessing symptom control for a 4-week period considering the frequency of asthma symptoms, night waking because of asthma, frequency of SABA^b^ use, and any activity limitation because of asthma; uses recognized screening, symptom control or numerical asthma control tools, and tracks peak flow measurement
Ability to self-track symptoms with or without peak flow	—^c^	Encourages patients to track symptoms and peak flow measurements
Risk factors for future exacerbations	Helps users identify the future risk of exacerbations	Helps users identify the risk of future exacerbations
Screens for comorbidities and education regarding managing them	Screens for comorbidities and assists patients with managing them	Screens for relevant comorbidities and educates patients on the management of these comorbidities
Inhaler technique with or without video	Provides education on appropriate inhaler techniques	Provides education on appropriate inhaler techniques with visual aids
Ability to record action plan	Provides an area for patients to keep and refer to their written action plan	Provides an area for patients to keep and refer to their written action plan
Reminder to engage with primary care	Reminds users to see their HCP^d^ for management and review of asthma	Provides reminders to users to see their HCP for management and review of asthma
—	Specifically provides suggestion to see an HCP if a patient is using only a SABA.	Specifically provides suggestion to see an HCP if a patient is using a SABA alone
Medication adherence	Prompts users to adhere to controller medications even when symptoms are infrequent	Prompts users to adhere to controller medications even when symptoms are infrequent
General asthma education	Provides knowledge on general asthma information, management of asthma, modifiable risk factors and strategies to address them, and when to see an HCP	Provides knowledge on general asthma information, management of asthma, modifiable risk factors and strategies to address them, when to see an HCP, and identification and management of comorbidities
Help with activating action plan	Provides advice on when to refer to a patient’s asthma action plan based on self-monitoring of symptoms or PEF^e^	Provides advice on when to refer to a patient’s asthma action plan based on self-monitoring of symptoms or PEF
—	Prompts patient to see the primary HCP if features of asthma exacerbation (symptoms and SABA use) are identified using the app	Prompts patient to see the primary HCP if features of asthma exacerbation (symptoms and SABA use) are identified using the app

^a^ACQ: Asthma Control Questionnaire.

^b^SABA: short-acting β-agonist.

^c^Recommendation identified by one reviewer but not the other.

^d^HCP: health care provider.

^e^PEF: peak expiratory flow.

### Identification, Screening, and Selection of Mobile Apps for Review

This review included both free and paid apps from the 2 most popular app stores in Australia across the iOS and Android operating systems: the Apple App Store (Apple) and Google Play Store (Google). Our published protocol outlines the steps taken for quality assurance [12]. Our approach for identifying these apps followed the approach used in similar studies [9-11]. Before commencing the initial search for apps, the reviewers ensured that the operating systems on the chosen smartphones were up to date. Each reviewer used different phones to assess the apps, but all updated the Android operating system (OS) to the Android 11 OS (Google) when reviewing apps from the Google Play Store. In the search bar of each store, we input the term *asthma*. Two reviewers (BR and DZ) independently searched both app stores on August 10, 2021, from Melbourne, Australia. After obtaining the results for this search term, each reviewer stored the information on an Excel (Microsoft Corp) spreadsheet (Multimedia Appendix 2). The reviewers then compared their results to ensure that they captured all the available apps.

For further evaluation of all the apps identified above, BR and DZ individually reviewed the app title, description, and attached photos and determined whether the app met all inclusion criteria and no exclusion criteria. An identified asthma app was included in the evaluation stage if all the following applied: (1) its primary role was related to asthma, (2) it was targeted to those with asthma, (3) it could be run on mobile phones, and (4) it was in English. Apps were excluded if any of the following applied: (1) they were not primarily related to asthma, (2) they were primarily targeted toward health care professionals (as stated in the product description), (3) they were not in English, and (4) they were targeted toward pediatric patients. This information was entered into an Excel (Microsoft Corp) spreadsheet for record-keeping (Multimedia Appendix 3). For further evaluation, all apps identified as meeting the above criteria were downloaded by a third reviewer (EP) who identified apps that did not install or function properly after downloading, eliminating them from the review. Finally, the last round of screening was conducted by the reviewer EP. In this round, duplications (ie, apps available on both stores), inaccessible apps, and “lite” version apps, where a pro version was available, were removed from the review. This process was comparable with similar reviews that examined the quality of mobile apps for diabetes self-management [15].

### App Evaluation and Data Extraction

#### Reviewer Training

A day-long training session was conducted before the initial data extraction. This training session was similar to the one performed by Gong et al [15] for their diabetes app review [15]. A step-by-step reference guide was created by the primary researchers to inform reviewers regarding how to complete the various frameworks and checklists involved in the study. This is provided in Multimedia Appendix 4 [3,4,16,17].

#### App Evaluation and Data Extraction Overview

An internet database was established on Qualtrics (Qualtrics International Inc) for data extraction. A total of 3 reviewers were involved in data collection. A web-based random team generator was used for all apps identified for further evaluation during the screening process so that each app was randomly allocated to 2 assessors (BR, DZ, or EP). The 2 assessors assigned to the app independently reviewed the in-store app description, downloaded the app, and used it for a minimum of 20 minutes to become familiar with all its functions [12]. The reviewers subsequently conducted the evaluations and entered the data into the Qualtrics database. Each reviewer performed this process individually without communicating their results to one another. There are 4 key aspects of the app evaluation and data extraction process, as summarized in the checklist provided in Multimedia Appendix 5 and the step-by-step guide to data collection in Multimedia Appendix 4.

#### Technical Information About the App

The first step in data collection involved gathering basic technical information about the app. The decision of which technical information to include was based on the MARS checklist and previous app review studies [7,9,15]. This was derived from publicly available information in the in-store app descriptions and in-app information sections. If required, the app developer’s website was used. The technical information collected included the app name, date of release, date of update, developer, developer affiliations, price, rating, number of ratings, platform or platforms, size of the app, and number of downloads. A checklist for this section is provided in Multimedia Appendix 5.

#### App Quality Assessment

The app quality assessment was completed using the MARS tool to objectively determine the quality of the apps selected. This scale has 4 separate domains that are assessed to evaluate mobile app quality. These domains are engagement, functionality, esthetics, and information quality [7]. A total of 19 items, each with a 5-point scale regarding quality in the 4 domains mentioned above, make up the MARS score [7]. This framework is presented in Multimedia Appendix 5. Reviewers completed this tool by entering the information into the Qualtrics (Qualtrics International Inc) checklist for each app. Once this was completed, the mean score for that domain and the overall MARS score were calculated for every app. Following the objective MARS section, there are several subjective questions to evaluate user satisfaction and the perceived impact of the app on the user’s knowledge, attitude, motivation to change, likelihood of change, and awareness of the importance of changing their asthma self-management [7]. These questions were answered by reviewers based on their experience using the app and their knowledge gained through the training sessions and clinical practice. Once the data were collected, the mean total MARS value and SD were calculated for each app.

The MARS tool assesses the presence or absence of 19 behavioral change techniques (BCTs). Although 93 types of BCTs are known, only the techniques outlined in MARS were assessed. This approach aligns with previous research, with a scope comparable with our review [7,10,15]. These techniques are outlined in Multimedia Appendix 4. To capture the presence of BCTs, an app was considered to have a BCT present, even if only 1 reviewer identified it. The median number of BCTs and the corresponding IQR of the apps was calculated using Stata (StataCorp) statistical software.

#### App Functionality

App functionality refers to what the app can do for a user and is an important marker of whether an app offers much benefit to users and the overall quality of the app. Although the MARS framework examines the overall quality of a mobile app, it focuses on the performance, ease of use, gestural design navigation, and navigation of the app [7]. Therefore, the Intercontinental Medical Statistics Institute for Health Informatics (IMS) Functionality Scoring System, henceforth known as the IMS functionality score, was used. This score was developed by the above institute and is based on 7 functionality criteria and 4 subcategories in the *record* functionality criterion. The IMS functionality score focuses on the scope of functions, including the ability of the app to inform, instruct, record, display, guide, remind or alert, and communicate information [18]. Each app was assessed for having or not having these functions and then given a total functionality score between 0 and 11 [18]. To capture the presence of all functionalities, an app was considered to have a functionality present even if one reviewer identified it. The mean, median, and IQR were calculated. The reviewers assessed each app for these functions and entered the data into Qualtrics (Qualtrics International Inc) database. This scoring system is presented in Multimedia Appendix 5.

#### Presence of App Features Consistent With Asthma Guidelines

As discussed above, key recommendations that could feasibly be incorporated into an asthma mHealth app were identified from the GINA guidelines. These recommendations, summarized in Table 1, were used to develop a more extensive checklist provided in Multimedia Appendix 5. The main functions of the app that we were interested in assessing in our checklist included asthma information, self-management skill training (including peak flow use, inhaler technique, and nonpharmacological strategies), monitoring of asthma symptoms, risk evaluation, and prompting (medication reminders, referring to action plan reminders, and suggestions for seeking health advice). Each of the selected apps was assessed using this checklist, and the data were entered into the Qualtrics (Qualtrics International Inc) database. To ensure consistency, an app was only assessed for the presence of the above function if both reviewers reached a consensus that the said feature was present. For single-reviewed those apps, the sole reviewer’s analysis was used to determine whether the app did or did not have the examined feature.

#### Quality Assurance, Data Management, and Data Analysis

Training was provided to all the researchers, and a handbook for reviewers was provided to ensure the quality of this research. Selected apps were allocated to reviewers using a web-based random allocation software, and 2 different major app databases were searched to reduce selection bias. The apps were independently reviewed by 2 reviewers to reduce the likelihood of bias affecting the results. A protocol was published to reduce publication bias and enhance the transparency of this study [12]. During app evaluation, all data were entered into either an Excel (Microsoft Corp) spreadsheet during the screening process or into the web-based Qualtrics (Qualtrics International Inc) database. These were stored on a cloud-based system that only the researcher team could access. Once the evaluation was completed, all data were downloaded for subsequent analysis. This analysis comprised a descriptive analysis and calculation of the mean and SD or median and IQR.

All data analyses were performed using Stata statistical software version 14 (StataCorp). Visual figures were generated using Excel (version 16; Office 365; Microsoft Corp).

## Results

### Identification, Screening, and Selection of Mobile Apps for Review

**Figure 1:**
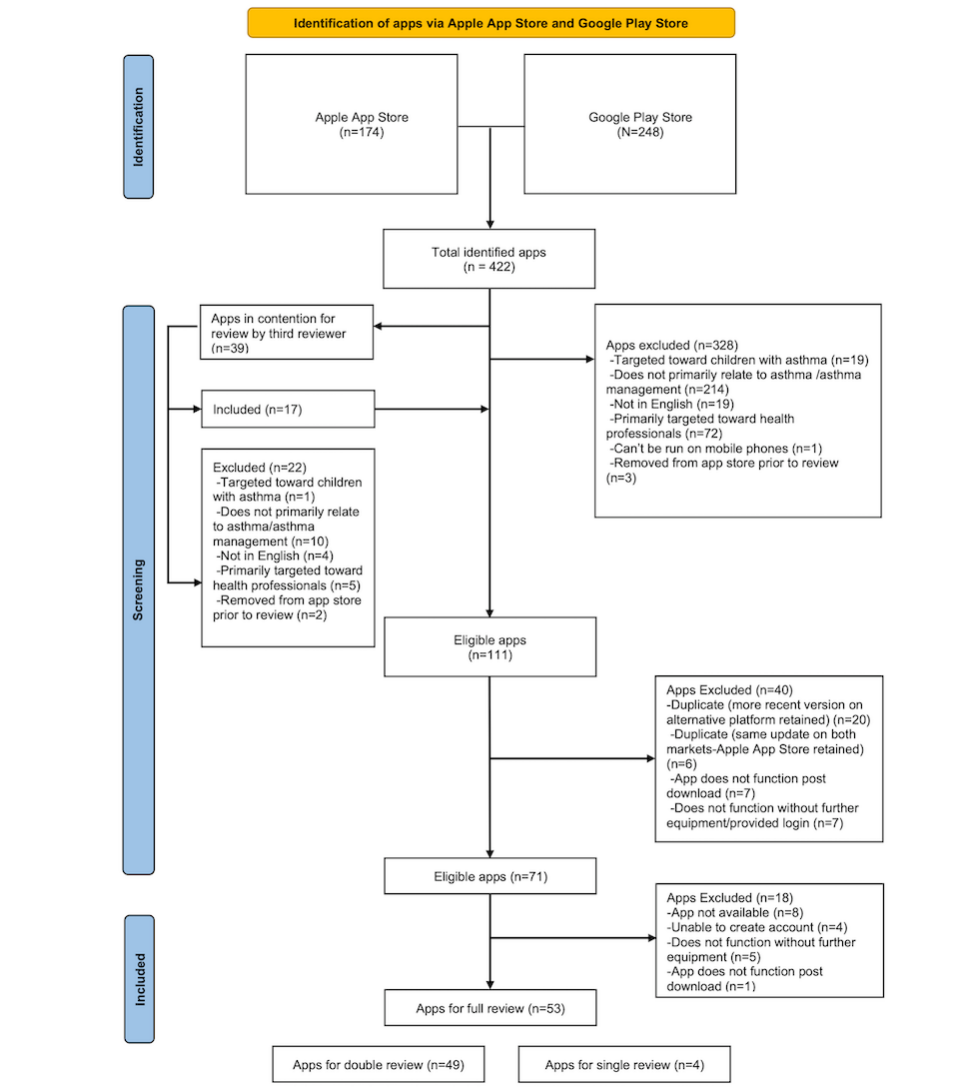
App screening process and results based on PRISMA (Preferred Reporting Items for Systematic Reviews and Meta-Analyses) guidelines.

The process and results of identifying, screening, and selecting mobile apps are shown in Figure 1. A total of 174 unique apps from the Apple Store and 248 unique apps from the Google Play Store were identified. These 422 apps were assessed by 2 reviewers (BR and DZ). In total, 94 apps met all inclusion criteria and no exclusion criteria, although there was a discrepancy between the reviewers’ opinions regarding the eligibility of 39 apps. A third reviewer (EP) identified 17 of these 39 apps as suitable for further review, which resulted in a total of 111 apps suitable for further assessment. A total of 40 apps were removed for reasons outlined in Figure 1. When a more recently updated app was available on 1 platform compared with the other, the older version was excluded from the review. When duplicate apps were available on both platforms and had been updated on the same date, the app from the Apple App Store was retained, whereas the app from the Google Play Store was excluded from the review. This was done to ensure consistency between reviewers and prevent skewing of results by assessing the same app twice. This resulted in 71 apps that were suitable for a complete assessment. Given the delay between the identification of apps and analysis, 18 of the above 71 apps were no longer assessable for the reasons outlined in Figure 1. A total of 4 apps were downloaded by 1 reviewer but not the other before they were removed from the market. This resulted in 49 apps assessed by 2 reviewers and 4 apps assessed by 1 reviewer (53 apps and 102 total reviews).

### Technical Information About the App

The technical information for each reviewed app can be found in Multimedia Appendix 2. Of the 53 apps assessed, 29 (55%) were from the Apple App Store, and 24 (45%) were from the Google Play Store. A total of 19 (36%) apps downloaded from the Apple App Store were also available on Google Play Store. As outlined above, apps available on both marketplaces were only downloaded from the Apple App Store and assessed on the iOS platform. The apps’ last date of update ranged from February 2016 to April 2022. A total of 26 (49%) apps were updated from January 2020. The mean app size was 46.33 MB, and the median app size was 27 MB (IQR 9.2 MB-47.38 MB). App developers were primarily technical companies (28 apps), health care or pharmaceutical companies (4 apps), or a combination of both (4 apps). Six apps were created by private individuals, 3 were created from research or clinical institutions and the remaining 8 were created from developers from a variety of other backgrounds. The number of app downloads ranged from 10 to >10,000. Of 53 apps, 24 (45%) apps had a published user rating, and the median rating was 4 out of 5 (IQR 2.9-4.9). The number of people who provided a rating ranged from 0 to 523, with a median of 2.5 ratings per app (IQR 1-20). Of 53 apps, 42 (79%) apps were completely free, 5 (9%) apps required users to pay to download, and 6 (11%) of the above free apps had the in-app ability to upgrade for a cost.

### Presence of Behavior Change Techniques

The total number of each BCT identified across the 53 apps assessed is demonstrated in Table 2. The most frequent BCT observed was *self-monitoring or tracking*, which was identified in 38 (72%) of the 53 apps. The next commonly identified BCTs were *information or education*, seen in 33 (62%) apps, and *advice, tips, strategies, or skills training*, seen in 26 (49%) apps. The average number of BCTs in a single app from those reviewed was 3.26 (SD 2.27). The median number of BCTs for the apps reviewed was 3 (IQR 1-4).

**Table 2:** Assessed behavioral change techniques and the number of apps found to use these techniques (n=53).

Behavioral change technique	Apps with this technique, n (%)
Information or education	33 (62)
Self-monitoring or tracking	38 (72)
Advice or tips or strategies or skills training	26 (49)
Assessment	17 (32)
Feedback	12 (23)
Self-efficacy	9 (17)
Model or demonstrate behavior	3 (6)
Rewards and self-rewards	6 (11)
Goal setting	5 (9)
Provide social support	3 (6)
Perceived risks	1 (2)
Model or demonstrate behavior	3 (6)
Action planning	11 (21)
Motivation	2 (4)
Motivational readiness	2 (4)
Mindfulness or meditation	1 (2)
Perceived benefit	1 (2)

### App Quality (MARS)

The mean MARS score for all reviewed apps was 3.05 (SD 0.54)**.** Of the 53 apps, 27 (51%) achieved a total MARS score of ≥3. A score of 3 on the MARS tool correlates to an “acceptable” quality app, <3 is inadequate or poor quality, and >3 represents a good or exceptional app [7]. Functionality was the highest rated MARS category with a mean score of 3.85 (SD 0.52), followed by esthetics with a mean score of 3.21 (SD 0.6). The information category had an average score of 2.78 (SD 0.83), and engagement had a mean score of 2.77 (SD 0.59). Table 3 shows the mean score for each of the 19 items on the MARS tool. Notably, the apps reviewed had higher scores in the gestural design, app description accuracy, and ease of use domains and lower scores in the evidence base, credibility, and entertainment domains. The mean scores for the quality and quantity of information were 3.21 (SD 1.95) and 2.68 (SD 1.63), respectively.

The final component of MARS allows reviewers to complete a subjective assessment of their opinions on the app. For double-reviewed apps, the mean value for the level of agreement for each domain was first calculated. Therefore, the number of apps will not be a whole number. This is summarized in Table 4 and demonstrates that there were few apps that reviewers would recommend to others, use >2 times in a 12-month period, or pay for. Only 8% (n=4) of the apps were rated >3 stars by the reviewers. For >50% of the apps, reviewers either disagreed or strongly disagreed that the app would impact the user’s knowledge, attitudes, and intentions to change or change the rate of asthma exacerbations. For <20% of the apps, reviewers either strongly agreed or agreed that the app would improve the user’s knowledge, attitudes, awareness, or intention to change behaviors to improve asthma self-management. However, reviewers either strongly agreed or agreed that 29% (n=15) and 27% (n=14) of apps would encourage users to seek further help in asthma management and reduce asthma exacerbations, respectively.

**Table 3:** Mean score for each category in the Mobile App Rating Scale (MARS) tool for the 53 assessed apps. Each category is assessed on a 5-point scale.

MARS category	Mean score for category
Entertainment	2
Interest	2
Customization	2
Interactivity	2
Target group	3
Performance	3
Ease of use	3
Navigation	3
Gestural design	4
Layout	3
Graphics	3
Visual appeal	2
Accuracy of app description	4
Goals	3
Quality of information	3
Quantity of information	2
Visual information	2
Credibility	1
Evidence base	1

**Table 4:** Results from the subjective assessment section of the Mobile App Rating Scale framework (n=53).

			Apps, n (%)
**Would you recommend this app to people who might benefit from it?**			
	Not at all, I would not recommend this app to anyone.	12 (23)	
	There are very few people I would recommend this app to.	18 (35)	
	Maybe, there are several people whom I would recommend it to.	18 (35)	
	There are many people I would recommend this app to.	4 (8)	
	Definitely, I would recommend this app to everyone.	0 (0)	
**How many times do you think you would use this app in the next 12 months?**			
	0	4(8)	
	1-2	28 (52)	
	3-10	7 (13)	
	11-50	14 (27)	
	>50	0 (0)	
**Would you pay for this app?**			
	No	34 (64)	
	Yes	18 (35)	
	Maybe	1 (2)	
**What is your overall star rating for the app?**			
	1 (one of the worst apps I have used)	10 (19)	
	2	20 (38)	
	3 (average)	18 (35)	
	4	3 (6)	
	5 (one of the best apps I have used)	1 (2)	
**Strongly disagree or disagree that the app will improve**			
	Awareness	40 (75)	
	Knowledge	37 (69)	
	Attitudes	32 (60)	
	Intention to change	32 (60)	
	Help seeking	27 (50)	
	Behavior change (reduce asthma exacerbations)	29 (54)	
**Strongly agree or agree that the app will improve**			
	Awareness	8 (15)	
	Knowledge	10 (19)	
	Attitudes	7 (13)	
	Intention to change	6 (12)	
	Help seeking	15 (29)	
	Behavior change (reduce asthma exacerbations)	14 (27)	

### IMS Functionality Score

Out of a potential 11 functionalities, an average IMS functionality score of 5.1 (SD 2.79) was achieved by the reviewed apps. The median number of functionalities identified was 5 (IQR 2-7). A total of 3 apps had 11 functionalities, although most apps had the ability to capture user-entered data (n=37, 70%), provide information in a variety of formats (n=35, 66%), and provide instructions to the user (n=33, 62%). Out of 53 apps, 7 (13%) apps had the ability to send alerts or propose behavioral interventions based on the collected data. The total number of apps that met these IMS functionality criteria is summarized in Table 5.

**Table 5:** Total number of apps meeting each of the Intercontinental Medical Statistics Institute for Health Informatics (IMS) functionality criteria (n=53).

IMS functionality	Apps containing this functionality, n (%)
Inform	35 (66)
Instruct	33 (62)
Record	37 (70)
Display	30 (57)
Guide	11 (21)
Remind or alert	27 (51)
Communicate	17 (32)
Collect data	37 (70)
Share data	26 (49)
Evaluate data	12 (23)
Intervene	7 (13)

### Presence of App Features Consistent With Asthma Guidelines

#### Knowledge

Table 6 outlines the results of evaluating the presence of information or knowledge identified as important based on the GINA guidelines. This details the number of apps where reviewer consensus was achieved, the number of apps that provided knowledge or did not provide knowledge in the subcategories, whether knowledge was individualized, and whether knowledge was based on evidence.

**Table 6:** Number of apps providing knowledge in asthma in the various relevant domains identified as important based on the Global Initiative for Asthma guidelines (n=53).

	Reviewer consensus achieved, n (%)	Apps that provided knowledge in this domain, n (%)	Apps that did not provide knowledge in this domain, n (%)	Apps that provided individualized knowledge, n (%)	Apps that provided evidence-based knowledge, n (%)
General asthma knowledge	45 (85)	10 (22)	35 (78)	0 (0)	7 (16)
Asthma medications	46 (87)	13 (28)	33 (72)	0 (0)	12 (26)
Exacerbation management	44 (83)	14 (32)	30 (68)	0 (0)	12 (27)
Asthma risk factors and triggers	46 (87)	11 (24)	35 (76)	0 (0)	11 (24)

#### Skill Training

The number of apps that provide skills training in peak flowmeter use, inhaler device use, recognizing and responding to asthma exacerbations, and nonpharmacological management to reduce asthma exacerbations are summarized in Table 7.

**Table 7:** Number of apps which provide the specific skill training in the areas identified as important in the Global Initiative for Asthma guidelines (n=53).

			Apps with reviewer consensus, n (%)		Apps that provide skill training, n (%)		Apps which provide personalized skill training, n (%)
**App provides general skill training in peak flowmeter use**			46 (87)		8 (17)		0 (0)
	Describes why and when to use peak flowmeter	0 (0)		5 (11)		0 (0)	
	Describes operational criteria for peak flowmeter	0 (0)		2 (4)		0 (0)	
	Demonstrates the use of peak flow meter through photos or videos	0 (0)		5 (11)		0 (0)	
**App provides general skill training in inhaler device use**			49 (93)		12 (25)		0 (0)
	Describes how to use a spacer	0 (0)		0 (0)		0 (0)	
	Demonstrates how to use a spacer through videos or photos	0 (0)		0 (0)		0 (0)	
	Demonstrates how to care for a spacer	0 (0)		0 (0)		0 (0)	
	Describes how to use common inhaler devices	0 (0)		0 (0)		0 (0)	
	Demonstrates how to use common inhaler devices through videos or photos	0 (0)		0 (0)		0 (0)	
**App provides general skill training in recognizing and responding to exacerbations**			39 (74)		11 (28)		3 (8)
	Encourages patients to monitor for signs of asthma exacerbation	0 (0)		9 (23)		1 (3)	
	Provide an area for asthma action plan	0 (0)		5 (13)		2 (5)	
	Specifically guides patients to use their asthma action plan	0 (0)		3 (8)		2 (5)	
	Provide information on how to use an asthma action plan	0 (0)		7 (18)		1 (3)	
	Prompts patient to see health care provider when required	0 (0)		7 (18)		0 (0)	
**App provides general skill training in nonpharmacological management strategies to reduce asthma exacerbations**			45 (85)		14 (31)		1 (2)
	Helps identify triggers that make symptoms worse	0 (0)		9 (20)		1 (2)	
	Advises avoidance of environmental smoke exposure	0 (0)		13 (29)		0 (0)	
	Advises avoidance of medications that can worsen asthma	0 (0)		2 (4)		0 (0)	
	Advises avoidance of occupation exposures	0 (0)		1 (2)		0 (0)	
	Advises on the avoidance of allergen exposure	0 (0)		13 (29)		0 (0)	
	Advises on avoidance of indoor or outdoor pollution	0 (0)		12 (27)		1 (2)	
	Advises on avoidance of emotional stress	0 (0)		4 (9)		0 (0)	
	Advises on regular moderate physical activity	0 (0)		6 (13)		1 (2)	

### App’s Ability to Track and Display Health Information

Table 8 demonstrates the results of the assessment of whether apps had the ability to track and display different aspects of a user’s key asthma information. All information is only related to apps where reviewer consensus was achieved or those that were reviewed by a single researcher. This demonstrates that most apps did not support tracking of all relevant asthma data, and for those that did, manual data input was the predominant entry method.

**Table 8:** Number of apps which allowed tracking and displaying of the specified asthma information identified as important from the Global Initiative for Asthma guidelines and by what means this information could be input into the app (n=53).

App’s ability to track and display users’ asthma information	Apps with reviewer consensus, n (%)	Apps that allow tracking and recording of data, n (%)	Apps with manual data input, n (%)	Apps that allow data entry through external sensors or devices, n (%)	Apps with the ability to create tables or graphs demonstrating trends or analysis of entered data, n (%)
Asthma symptoms	51 (96)	19 (37)	19 (37)	1 (2)	14 (27)
Night waking because of asthma	49 (92)	4 (8)	4 (8)	0 (0)	2 (4)
Activity limitation because of asthma	47 (89)	3 (6)	3 (6)	0 (0)	3 (6)
Peak flow meter values	50 (94)	20 (40)	17 (34)	4 (8)	20 (40)
SABA^a^ use	46 (87)	14 (30)	14 (30)	3 (7)	10 (21)
Preventive medication adherence	48 (91)	11 (23)	11 (23)	3 (6)	8 (17)

^a^SABA: short-acting β-agonist.

### App’s Ability to Provide Prompts or Reminders

Table 9 demonstrates the results of the assessment of whether the apps could provide reminders or prompts on the areas deemed relevant from the review of the GINA guidelines, as outlined in the Methods section. Overall, there were few apps that provided reminders or prompts to users, with only 9 (20%) out of 53 apps providing a reminder to use a person’s daily preventive medication. Few apps prompted users to assess the severity and frequency of their asthma symptoms or to seek health advice based on the data they entered.

**Table 9:** Number of apps that provided reminders and prompts on the specified asthma features chosen based on Global Initiative for Asthma guidelines (n=53).

App provides reminders or prompts on asthma features	Apps with reviewer consensus, n (%)	Apps that provide reminders or prompts, n (%)	Apps where reminders or prompts can be individualized, n (%)
Assessing asthma symptoms for the last month	50 (94)	2 (4)	1 (2)
Appointment with physicians	47 (89)	1 (2)	1 (2)
Performing peak flow test	50 (94)	5 (10)	4 (8)
Preventive medication adherence	45 (85)	9 (20)	8 (18)
Checking the date of expiry and dosage of inhalers	51 (96)	0 (0)	0 (0)
Warning of changing health data (ie, very frequent exacerbations)	53 (100)	0 (0)	0 (0)
Seeking urgent health advice based on the health data the user inputs into the app	52 (98)	2 (4)	2 (4)

### Other App Information

Table 10 summarizes the other important features assessed during this review. Only 1 app allowed the user to make an appointment with a physician. Of import, only 5 (10%) of the apps were identified as having an area where users could keep a record of their asthma action plan. Most of these (4 apps) allowed the user to type in their action plan, and 1 app allowed users to upload a photo of their action plan. Only 1 app was identified using recognized asthma screening tools to assess patient’s current asthma symptoms and severity.

**Table 10:** Summary of number of apps containing further features and content identified as important (n=53).

			Reviewer consensus, n (%)		Apps with this feature, n (%)		Apps without this feature, n (%)
**Make appointment with physicians**			53 (100)		1 (2)		52 (98)
	For web-based consultation	0 (0)		0 (0)		53 (100)	
	For face-to-face consultation	0 (0)		1 (2)		52 (98)	
**App provides an area for patients to keep record of their asthma action plan**			52 (98)		5 (10)		47 (90)
	Can type in action plan	0 (0)		4 (8)		48 (92)	
	Can upload a photo of action plan	0 (0)		1 (2)		51 (98)	
The app includes social forums or blogs that promote peer-support and communication among asthma patients			50 (94)		1 (2)		49 (98)
**The users could send recorded data to others**			44 (83)		11 (25)		33 (75)
	To physicians	0 (0)		11 (25)		33 (75)	
	To each other	0 (0)		9 (21)		33 (75)	
**The app could help the users to evaluate the risk of having future asthma exacerbations**			44 (83)		2 (5)		42 (96)
	Using a validated scoring system	0 (0)		0 (0)		44 (100)	
	Yes, but not using a validated scoring system	0 (0)		2 (5)		42 (96)	
The app could guide the users to find out the closest pharmacy, hospital, or clinic			51 (96)		0 (0)		51 (96)
**The app uses recognized screening, symptom control and numerical asthma control tools**			46 (87)		1 (2)		45 (98)
	Yes, all 3	0 (0)		0 (0)		46 (100)	
	Yes, screening tool	0 (0)		0 (0)		46 (100)	
	Yes, symptom control tool	0 (0)		0 (0)		46 (100)	
	Yes, numerical asthma control tools	0 (0)		1 (2)		45 (98)	
**The app allows users to connect to wearable technology**			53 (100)		8 (15)		45 (85)
	Smartwatch	0 (0)		0 (0)		53 (100)	
	Activity sensor (eg, Fitbit)	0 (0)		0 (0)		53 (100)	
	Smart peak flowmeter	0 (0)		4 (8)		49 (93)	
	Handheld spirometer	0 (0)		3 (6)		50 (94)	
	Oximeter	0 (0)		2 (4)		51 (96)	
	Other	0 (0)		6 (11)		47 (89)	

## Discussion

### Principal Findings

This review aimed to evaluate the quality and functionality of asthma apps and their consistency with international best practice guidelines. We conducted a comprehensive review of 422 asthma apps available on the Australian App Store and Google Play Store, of which we selected 53 apps that met our inclusion and exclusion criteria for detailed analysis. The most common reason for app exclusion was that they were not primarily related to asthma. Most apps were developed by technical companies rather than health care facilities and clinical or research institutes. This lack of involvement of practicing experts in the field of health care is concerning, as these apps are primarily related to the management of chronic health conditions. Apps that involved a clinician during the designing phase demonstrated a greater ability to facilitate behavioral change than those that did not [19]. We believe that in the future, asthma mHealth apps should be developed in consultation with health care professionals and organizations to ensure that they meet an appropriate standard.

Our review revealed that most asthma apps do not use key BCTs that can promote behavioral changes through feedback, goal setting, and rewards. Instead, the most commonly used BCTs were self-monitoring or tracking, education, and advice. This is consistent with other studies and demonstrates a potential area where asthma apps could be improved in the future [19,20]. Functionalities describe what an app can do for its user and are an important marker of whether an app offers much benefit to users. Our review demonstrated that although basic functionalities, such as informing, reading, collecting, instructing, and displaying, were prominent, more complex functionalities were lacking. The ability to evaluate and intervene based on the app information entered is not present in most apps. Creating apps with this functionality could, for example, guide patients to see their health care provider based on the data they are entering, such as excessive short-acting β-agonist use or asthma symptoms. This feature was demonstrated in the Asthmahub app from NHS Wales, one of the higher-rated apps on assessment.

Our evaluation using the MARS tool showed that the asthma mHealth apps performed poorly in the information and engagement domains compared with the functionality and esthetics categories. These findings are consistent with previous asthma app reviews that showed poorer results in these categories [9]. Only half of the apps achieved an acceptable standard, and even fewer achieved a dual rating average >4, indicating a “good-quality” app. These apps were Kiss myAsthama, Asthmahub, and AioCare Patient. Acceptable or good ratings for subcategories such as app credibility, evidence base, and entertainment were particularly lacking in the apps we assessed. In contrast to this lack of evidence-based content identified through app assessment with the MARS framework, assessment with our checklist found that the knowledge presented in apps was largely based on evidence. “Evidence-based” in the MARS checklist refers to whether an app has undergone a clinical trial. By contrast, in our checklist we refer to “evidence-based” as that the information presented in the app was factual and in alignment with our clinical knowledge of asthma and the GINA guidelines. This explains the contrasting results between the MARS framework and our checklist for “evidence-based” knowledge. Although information was often factual and based on guidelines, the apps had not undergone clinical trials. The subjective star rating provided by reviewers was rarely >3, whereas the mean user rating was 3.56 out of 5, indicating a discrepancy between the perspectives of the reviewers and the user. This discrepancy in user ratings and reviewers’ perspectives has been demonstrated in similar app reviews in the past [9]. We propose that this could be because of our health care background bias when assessing the apps, even though we were attempting to assess apps from the perspective of a patient with asthma, or because we were approaching the review with a critical lens following an objective app assessment, a different mindset from the usual user.

Our review demonstrated that asthma mHealth apps do not contain key features consistent with international best practice guidelines for asthma management. Few apps contained important information regarding asthma and asthma management, and even when provided, they were not individualized to the user. Personalization is a key part in the management of asthma, with an individual’s triggers, baseline respiratory function, and inhaler device being common things we assess for, educate on, and consider when managing asthma in the community. Furthermore, personalized therapeutic management is a key aspect of asthma management, as outlined in the GINA guidelines [4]. There is also an expectation from previous research that personalized technological interventions may lead to better health outcomes, although this has not yet been specifically demonstrated for asthma mHealth apps [21]. The NHS Wales AsthmaHub app provides one solution to this problem by starting with the creation of a profile in which the user answers questions regarding asthma triggers, control, and medications. This allows the app to provide information targeted toward the user. Skill training in a field can be achieved by apps through written and visual information to teach users how to use peak flow meters, inhalers, and nonpharmacological strategies to manage asthma and asthma exacerbation. These are skills that the GINA guidelines advocate patients to learn and become proficient in, with the support of their health care provider [4]. Training in the use of inhaler devices, spacers, and peak flow meters, all of which in our experience, patients can inadvertently misuse, is lacking in most apps and is something that we feel is key to be included in an asthma mHealth app. Although not essential for all inhaler types, such as dry-powder inhalers, spacers are considered an essential part of using metered-dose inhaler preventer and reliever medications. Therefore, we believe it is reasonable that a high-quality asthma mHealth app should include advice to the users regarding the use of spacers, particularly given the prevalence of salbutamol or albuterol metered-dose inhaler use among those with asthma. No apps could individualize this training to the user, and given the magnitude of devices on the market and the difference in how they work, this represents a deficiency. In total, 40% (n=20) of the apps could record and display peak flow meter values and asthma symptoms. Peak flow is representative of worsening airway obstruction or asthma. If this value is reducing, it can indicate that a person’s asthma may be on the precipice of or in the middle of an exacerbation, and as such, it can be a valuable metric for patients and clinicians to monitor. Other key metrics that could be tracked were lacking, such as the amount of short-acting β-agonists and adherence to preventive medications. It is important for these metrics to be recorded, as excessive SABA use can indicate an asthma exacerbation or poorly controlled asthma and should prompt a review by a health care professional. Subtler signs of poor asthma control, such as activity limitation or night waking because of asthma, were rarely recorded and represented missed opportunities. Prompting and reminding users to do something is a basic functionality that is largely underutilized by apps. Chronic asthma management involves remembering to consume daily inhaler medications, assessing symptoms, and regular interactions with a health care provider [4]. All these tasks lend themselves to a reminder from an app to assist with asthma management, and their absence from most apps is a missed opportunity.

Asthma action plans are a key aspect of contemporary patient self-management of asthma [4]. The ability to quickly refer to this plan on a user’s digital device should be easy to include; however, most apps lack this feature. We see this as a significant deficiency and missed opportunity. Few apps use validated asthma symptom scoring systems for users to assess their symptoms and risk of exacerbation. Wearable technology and external sensors are a growing medium through which data related to a patient’s asthma status can be captured, yet only few apps use this technology. When external sensors are used, they are often smart spirometers (such as in AioCare patient) or peak flow meters, both of which carry extra costs and may not be palatable to all users. No apps were found to be linked to smartwatches or activity sensors, the use of which is becoming more prominent [22]. With the availability of pulse oximetry in smartwatches, this may be a new method for asthma apps to collect crucial data in the future.

Overall, we determined the quality of apps to be average at best, with many lacking features consistent with international best practice guidelines. Three apps achieved a MARS rating of >4. These were Kissmyasthma, Asthmahub, and AioCare patient. Their alignment of these apps with international best practice guidelines was mixed, with Aiocare patients not having many of the functions deemed important by the guidelines. The Kissmyasthma and Asthmahub apps had great amounts of information related to asthma, consistent with the guidelines. Asthmahub stood alone in having these features and many features to support skill training and track and record information; however, its prompting or reminding functions were minimal. Notably, both these apps that ranked higher in MARS and our checklist were developed by health services or medical research centers (NHS Wales, the University of Sydney, The University of Melbourne, and the Woolcock Institute of Medical Research). All 11 functionalities from the IMS scale were identified in Asthmahub and Aiocare patients, and Kissmyasthma had 6 functionalities, which were above the mean identified. A total of 6 to 8 BCTs were identified in these apps, which, although greater than the median number of BCTs, still did not include several potential BCTs.

### Comparison With Previous Work

Prior studies that have conducted these assessments have primarily focused on evaluating the quality and functionality of apps using the MARS framework [9-11]. From a review of the literature for the past 5 years, only 2 prior studies were found to have conducted some sort of assessment of the alignment of apps with asthma self-management principles. Both these studies only looked at free apps, eliminating a number of apps from the review [11,23]. Data collection for both reviews occurred years ago [11,23]. In the rapidly developing marketplace of mobile apps, a number of new apps have been released during this time. Our review examined both free and paid apps, and provided an updated assessment, given that our data collection took place in 2022. Furthermore, Househ et al [23] did not assess apps from the Apple App Store, focusing only on the Google Play Store, and therefore, did not fully assess the breadth of available English-language apps in the marketplace. These authors evaluated whether apps included or did not include information consistent with GINA guidelines as per a checklist created by 1 author [23]. This was limited to asthma information and education and did not include further features, such as the ability of an app to track information, provide asthma skill training, or personalize information. This review also did not examine the overall app quality using the validated MARS framework [23].

Our review benefits from having 2 independent clinicians review the guidelines to establish all GINA self-management recommendations that can be feasibly incorporated into an mHealth app and review app quality using the MARS framework. Furthermore, we examined not only the presence of information, but also the presence or absence of the ability to track asthma symptoms and provide reminders and skill training, as well as all features derived from the GINA guidelines provided in Multimedia Appendix 5. As part of their app review, Tan et al [11] established a framework for assessing the alignment of mHealth apps with the theoretical principles of self-management of allergic rhinitis or asthma [11]. A total of 6 asthma self-management principles were identified based on a literature review and author consensus [11]. Our review has taken this a step further, specifically deriving self-management principles from the international best practice GINA guidelines and creating a more extensive checklist based on these principles. The inclusion of paid apps, the creation of an asthma self-management principle checklist derived from international best practice guidelines, and the up-to-date nature makes our study unique.

### Limitations

This study has several limitations. First, we limited it to English-language apps available from 2 app stores in Australia. Although these stores make up a significant portion of the market, other stores do exist, such as the Blackberry store, which were not included in the review. It is also expected that some apps available on the Australian marketplace may not be available on international marketplaces, whereas some apps available internationally may not be available on the Australian marketplace, and thus not included in the review. Although the operating systems for phones used by reviewers were updated on the same day to ensure that the same OS was on each smartphone, the phones themselves were different models. This may have affected the user or reviewer experience to an unknown degree, reducing standardization in this study. A total of 4 apps were reviewed by 1 researcher only as they were removed from the market or were not available to the second reviewer by the time they tried to assess it. This may skew some results, although previous studies have only single-reviewed apps; therefore, the fact that the most apps in this study are double-reviewed is a strength of this research.

Furthermore, we only assessed the presence or absence of those BCTs embedded in the MARS checklist. This limited the number of BCTs that we assessed; however, reviewing the presence or absence of all 93 BCTs was outside the scope of this review and our protocol and requires further research.

This evaluation was researcher-based, and thus does not reflect the real assessment from a patient’s perspective. Future research should also include people with asthma to determine their responses to the quality and functionality of these apps.

### Conclusions

Currently, there is a lack of high-quality asthma apps aligned with international best practice asthma guidelines. Most apps do not provide patients with important asthma information, skills training in key aspects of asthma management, recording and tracking relevant data, or reminders regarding asthma control. The lack of interaction with smart technology or use of asthma action plans are significant flaws that merit attention in future apps. Few BCTs or in-depth functionalities have been deployed to engage and re-engage with users or generate meaningful behavioral modifications. Again, we see that app designers are typically skilled in providing an esthetically pleasing functional app but lack skills in providing engagement and information, as assessed in the MARS tool. Future app development should target the key features identified in this study as currently lacking. Furthermore, not only are high-quality asthma mHealth apps lacking, there are minimal robust clinical trials examining the use of these apps and their effect on patient outcomes. Further research in this area will be valuable to determine the true clinical utility of these apps.

A wide spectrum of technological quality, accuracy, and breadth of health information was seen among available apps. Given this spectrum of poor-to high-quality apps in an unregulated market, it is unlikely that future guidelines or health professionals will be able to make generic recommendations to patients regarding asthma mHealth app use and instead will need to make targeted recommendations about specific apps. Guidelines that incorporate reviews such as this review that identify apps known to be of high quality, such as Asthmahub, AioCare patient, and Kissmyasthma, will be an important future resource for general clinicians navigating the ever-expanding mHealth app market for chronic diseases.

## Appendix

Multimedia Appendix 1 Completed PRISMA (Preferred Reporting Items for Systematic Reviews and Meta-Analyses) checklist.

Multimedia Appendix 2 App technical information.

Multimedia Appendix 3 Data from initial app review process to identify apps that met inclusion but not exclusion criteria.

Multimedia Appendix 4 Stepwise guide on the key steps of data extraction and evaluation for reviewers to follow.

Multimedia Appendix 5 Data extraction forms (technical information, Mobile App Rating Scale, Intercontinental Medical Statistics functionality score, and asthma assessment checklist).

## Abbreviations

BCT: behavioral change technique
GINA: Global Initiative for Asthma
IMS: Intercontinental Medical Statistics Institute for Health Informatics
MARS: Mobile App Rating Scale
mHealth: mobile health
PRISMA: Preferred Reporting Items for Systematic Reviews and Meta-Analyses
